# Using Injury Severity Score and Abbreviated Injury Score to Determine Venous Thromboembolism Risk

**DOI:** 10.7759/cureus.5977

**Published:** 2019-10-23

**Authors:** Timothy Hereford, Carol Thrush, Mary K Kimbrough

**Affiliations:** 1 Orthopaedic Surgery, University of Arkansas for Medical Sciences, Little Rock, USA; 2 Surgery, University of Arkansas for Medical Sciences, Little Rock, USA; 3 Trauma Surgery, University of Arkansas for Medical Sciences, Little Rock, USA

**Keywords:** trauma, dvt, vte, injury severity score, abbreviated injury score, venous thromboembolism, pe, iss, ais

## Abstract

Background

Venous thromboembolisms (VTEs) continue to be a leading cause of death among trauma patients. Predicting which patients will develop a VTE can be difficult. This study investigated whether the Injury Severity Score (ISS) could be used in conjunction with the Abbreviated Injury Score (AIS) to assess a trauma patient’s risk for subsequent VTE development.

Materials and Methods

Participants were found by querying a trauma center registry. There were 2,213 patients included for evaluation. The patients were categorized based on their ISS and the anatomical region with the greatest injury (determined by the AIS). Odds ratios for developing VTEs were calculated for each ISS category.

Results

The results showed that in most categories VTE risk increased as ISS increased. Patients with trauma to their head/neck, chest, or extremities with ISS values of 21 or greater were all at significantly increased risk for VTE development. Patients in these categories with an ISS less than 21 seemed to have little or only moderately increased odds of developing a VTE, although these values were not statistically significant. Patients with abdominal trauma were at increased risk even with ISS values of 11-21.

Conclusion

Trauma to the head/neck region, chest, and extremities (including pelvis) with ISS of 21 or higher had significantly increased odds of developing a VTE. Patients with abdominal trauma of any severity appeared to have increased odds of developing a VTE.

## Introduction

Venous thromboembolisms (VTEs) continue to be a constant and costly problem in the United States and abroad. VTEs are especially problematic in trauma patients. Deep vein thrombosis (DVTs) often occur asymptomatically and may later present as a pulmonary embolism (PE). PE is the third most common cause of death in trauma patients who survive the first 24 hours [1], and there is no way to accurately predict which trauma patients will develop a VTE. Trauma physicians must weigh the severity of the injury, the presence of risk factors, and clinical suspicion to determine if a patient’s risk for VTE outweighs the risk of anticoagulation therapy. Injury scoring systems, such as the Abbreviated Injury Score (AIS) and the Injury Severity Score (ISS), are often utilized to help assess the severity of the patient’s trauma. Studies have shown that higher ISS equate to higher VTE risk. Azu et al. studied 10,150 trauma patients and found that as ISS increased, VTE incidence increased as well [2]. There have also been studies that evaluated the AIS in relation to VTE risk. Paffrath et al. did a retrospective review of 7,937 trauma patients and found that patients with an AIS greater than or equal to 3 were at significantly greater risk for VTEs [3]. There have not been any studies, however, that have looked at the ISS in conjunction with the AIS to see if the two correlated together could be used to assess a patient’s VTE risk. The purpose of this exploratory study was to investigate whether a patient’s overall ISS could be used in combination with the injury pattern (determined by the AIS) to give a VTE risk assessment with the goal of creating a better screening tool for identifying patients at high risk for developing VTEs.

## Materials and methods

Participants were identified by a retrospective search on all trauma patients who were admitted from April 13, 2014 through July 31, 2015 at a medium-sized trauma center in the mid-south (average of 2,000 trauma patients per year during this time period). The start date was chosen based on the availability of the hospital’s electronic medical record system archive. The closing date was chosen because this was the most current data available at the time of the study. During this time period, 2,761 patients were entered into the trauma registry. A classification system was created that categorized patients by their ISS and the anatomical region with the highest AIS. The regions were head/neck, face, chest, abdomen, extremities (including pelvis), and external (skin). Patients who had a tie for the anatomical region with the highest AIS were excluded. This was done so that each anatomical region could be evaluated individually for VTE risk while minimizing crossover into other categories. After these exclusions, there were 2,213 trauma patients who remained for analysis (see Table 1). Patients were grouped into four ISS categories: 1-10, 11-20, 21-30, and 31 or greater. There were a total of 29 VTE events in this patient population, which is an overall VTE incidence of 1.31% (see Table 1). The odds ratio (OR) was calculated for each ISS category in each anatomical region and for the anatomical region overall. To calculate the OR, the ratio of VTEs in each ISS category was divided by the overall VTE rate: 

$OR = (VTEs\, in\, ISS\, Category \div Total\, Patients\, in\, ISS\, Category) \div (Total\, VTEs \div Total\, Patients)$

A 95% confidence interval and p-value were also calculated using MedCalc statistical software (Ostend, Belgium). 

**Table 1:** Total Number of Trauma Patients and Venous Thromboembolisms by Anatomical Region and Injury Severity Score VTE = venous thromboembolism; ISS = Injury Severity Score

ISS	Head/Neck	Face	Chest	Abdomen	Extremity	External	Totals
	Total Number of Trauma Patients						
1-10	409	103	87	70	497	388	1,554
11-20	154	3	151	25	66	4	403
21-30	83	0	42	18	4	5	152
31+	55	0	25	21	2	1	104
Total	701	106	305	134	569	398	2,213
	Total Number of VTEs						
1-10	2	0	0	1	3	0	6
11-20	1	0	3	2	1	0	7
21-30	4	0	2	1	1	0	8
31+	6	0	2	0	0	0	8
Total	13	0	7	4	5	0	29

## Results

The results of the data analysis can be found in Table 2. In almost every category, the VTE OR increased as the ISS increased. Several categories had results that were statistically significant. In the head/neck region, the OR for ISS category 21-30 was 3.68 (confidence interval [CI] 1.26-10.7) and for category 31 or greater, it increased to 8.32 (CI 3.32-20.9). In the chest region, the ISS category 31 or greater had an OR of 6.10 (CI 1.38-27.0). In the extremities group, the ISS category 21-30 had an OR of 19.1 (CI 2.01-176.0). In the abdominal region, the ISS category 11-20 had an OR of 6.10 (CI 1.38-27.0). There were no VTEs that occurred in the face region or external injury. 

**Table 2:** Odds Ratio for Venous Thromboembolism by Anatomical Region and Injury Severity Score VTE = venous thromboembolism; ISS = Injury Severity Score; CI = confidence interval; OR = odds ratio

	ISS Group	Total Patients	Total VTE	OR	CI (95%)	p-value
Head/Neck	1-10	409	2	0.373	0.089-1.57	0.179
	11-20	154	1	0.496	0.067-3.66	0.491
	21-30	83	4	3.68	1.26-10.7	0.017
	31+	55	6	8.32	3.32-20.9	<0.0001
	Total	701	13	1.42	0.732-2.74	0.302
Chest	1-10	87	0	-	-	-
	11-20	151	3	1.52	0.457-5.03	0.500
	21-30	42	2	3.63	0.840-15.7	0.084
	31+	25	2	6.10	1.38-27.0	0.017
	Total	305	7	1.75	0.761-4.03	0.188
Abdomen	1-10	70	1	1.09	0.146-8.12	0.933
	11-20	25	2	6.10	1.38-27.0	0.017
	21-30	18	1	4.24	0.548-32.8	0.167
	31+	21	0	-	-	-
	Total	134	4	2.28	0.789-6.57	0.128
Extremities	1-10	497	3	0.461	0.140-1.52	0.203
	11-20	66	1	1.16	0.155-8.62	0.887
	21-30	4	1	19.1	2.01-176.0	0.0093
	31+	2	0	-	-	-
	Total	569	5	0.671	0.258-1.74	0.411

## Discussion

The results of this study show a correlation with the findings of previous studies. As ISS increased, the VTE incidence increased. Moderate-to-severe trauma to the head/neck, chest, abdomen, and extremities all resulted in higher odds of developing a VTE. Studies have shown that trauma to these regions is associated with and increased risk for VTEs [1-3]. One reason for this is that these groups of patients often do not get immediate DVT prophylaxis until their injuries are stabilized due to risks of excessive blood loss. Interestingly, VTE risk was increased most dramatically in patients with an ISS of 21 or greater. In the lower ISS categories (1-10 and 11-20), the data seem to indicate there is little, or perhaps moderately, increased risk for developing a VTE. Patients with less severe injuries typically are not immobilized or hospitalized for long periods of time, both of which are risk factors for VTE. However, in these lower ISS categories, almost none of the OR values were statistically significant so more research is needed. Abdominal trauma was the only exception to this where ISS category 11-20 had a statistically significant OR of 6.10. One proposed possibility for the higher VTE risk for comparably moderate abdominal trauma is that abdominal trauma often requires blood transfusions even for less severe injuries. It has been shown that blood transfusions are a significant risk factor for developing a VTE [4],^ ^although this relationship was not explored in this study. Although the ISS category 21-30 for the abdominal region did not have significant results (OR 4.24, CI 0.548-32.8), it is possible that with more data, a significant increase in VTE risk would be found in this category as well. Patients with severe injuries in the extremity region had the highest OR for developing a VTE (OR 19.1). This region includes patients with pelvic injury, which is known to be a major risk factor for VTEs [1]. 

There were several limitations to this study. The higher ISS categories (above 20) had small sample sizes, which reduced the power of the study. Also, patients who had a tie for AIS in multiple regions were excluded from the OR analysis, which reduced the sample size even further. Survivor bias should also be considered. It is possible that some patients with severe injuries did not live long enough to develop a VTE. It is also possible that some VTEs went undiagnosed. Many of the findings could also be influenced by institutional-specific patient care practices. Results may vary depending on the anti-coagulation and DVT prophylaxis protocols at different institutions. Even so, this exploratory study could easily be replicated at other institutions or using national databases to verify the findings.

## Conclusions

Our results indicate that trauma to the head/neck region, chest, and extremities (including pelvis) with an ISS of 21 or higher had significantly increased odds of developing a VTE. Patients with an ISS less than 21 seemed to have little or only moderately increased odds of developing a VTE in most categories, although these values were not statistically significant. Patients with abdominal trauma of any kind seemed to have increased odds of developing a VTE; however, it is unclear if this increased risk is related to the anatomical region or to other factors. One of the goals of this study was to create a screening protocol that could be used to help identify trauma patients who are at especially high risk of developing a VTE. With further studies to verify our findings, this screening tool could alert the physician to consider more aggressive VTE screening and help physicians balance whether the risks of developing VTE outweigh the risks of earlier institution of prophylactic treatment.
